# An Unusual Presentation of Valentino Syndrome With a Perforated Gastric Ulcer as a Cause of Abdominal Pain: A Case Report

**DOI:** 10.7759/cureus.34845

**Published:** 2023-02-10

**Authors:** Mauricio Gutierrez-Alvarez, Santiago Vallarta, Rodrigo Cruz, Victor Visag-Castillo

**Affiliations:** 1 Department of General Surgery, Medica Sur, Mexico, MEX; 2 Department of Transplants, Instituto Nacional de Ciencias Médicas y Nutrición Salvador Zubirán, Mexico, MEX; 3 Department of Transplants, Hospital General de Mexico, Mexico, MEX

**Keywords:** abdominal pain, emergency, appendicitis, gastric perforation, gastric ulcer, valentino’s syndrome

## Abstract

Numerous pathologies can cause abdominal pain; thus, the surgeon's job is to precisely identify any pathologies that may require surgery and endanger the patient's life. Perforation of a gastric or duodenal ulcer associated with a clinical picture of acute appendicitis is known as Valentino syndrome (VS). To our knowledge, there are 22 cases of VS reported in the literature. We describe the clinical case of a 53-year-old female patient with abdominal pain in the right iliac fossa who came to the emergency room. A plain tomography was performed, which found free intraperitoneal fluid and free subdiaphragmatic air. Therefore, a laparotomy was performed, revealing a gastric perforation. VS is a rare pathology and when not recognized and managed properly, it can increase patients' mortality.

## Introduction

Abdominal pain is an entity caused by many pathologies. The surgeon's task is to be able to accurately diagnose pathologies that are potentially surgical and that may put the patient's life at risk, it is of utmost importance for emergency physicians and surgeons to take into account Valentino syndrome (VS) as a differential diagnosis for abdominal pain in the right iliac fossa. VS refers to the perforation of a gastric or duodenal ulcer associated with a clinical picture of acute appendicitis [1]. This syndrome is named after Rudolph Valentino, a movie star who underwent surgery for acute appendicitis at the Polytechnic Hospital of New York in 1926. After the surgical procedure, he developed peritonitis, dysfunction of multiple organs and systems, and died after several days. The autopsy revealed a perforated gastric ulcer, the gastric content reaching the right paracolic gutter causing peritoneal irritation simulating a picture of acute appendicitis [2,3]. The pathophysiology of this entity involves the gastrointestinal liquid secondary to gastric or duodenal perforation running through the right paracolic gutter to the ipsilateral iliac fossa, irritating the peritoneum, thus simulating a picture of acute appendicitis [4] and later even reactive appendicitis. Twenty-two cases of VS have been reported in the literature [5].

## Case presentation

**Figure 1:**
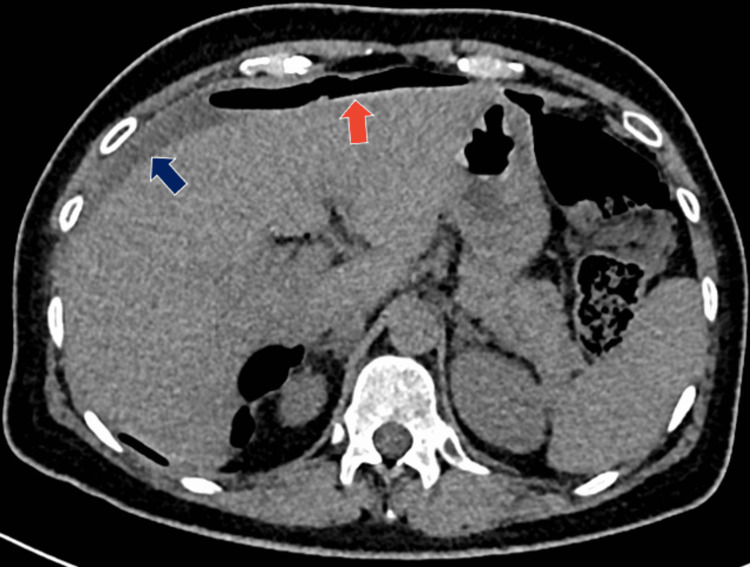
Red arrow indicates free air in the abdomen, and the blue arrow indicates free fluid.

**Figure 2:**
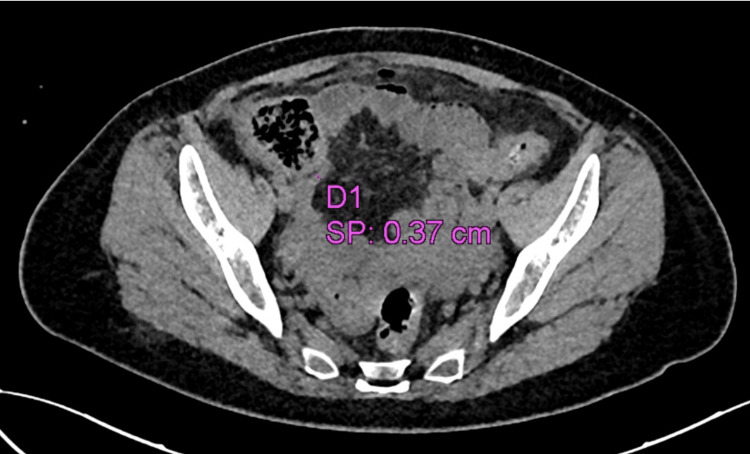
Cecal appendix of 0.37 cm

**Figure 3:**
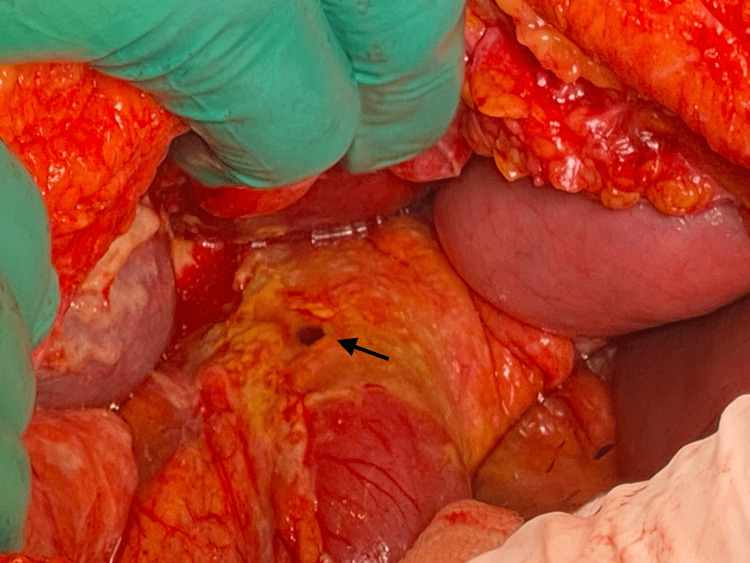
Perforation in pre-pyloric area (arrow)

**Figure 4:**
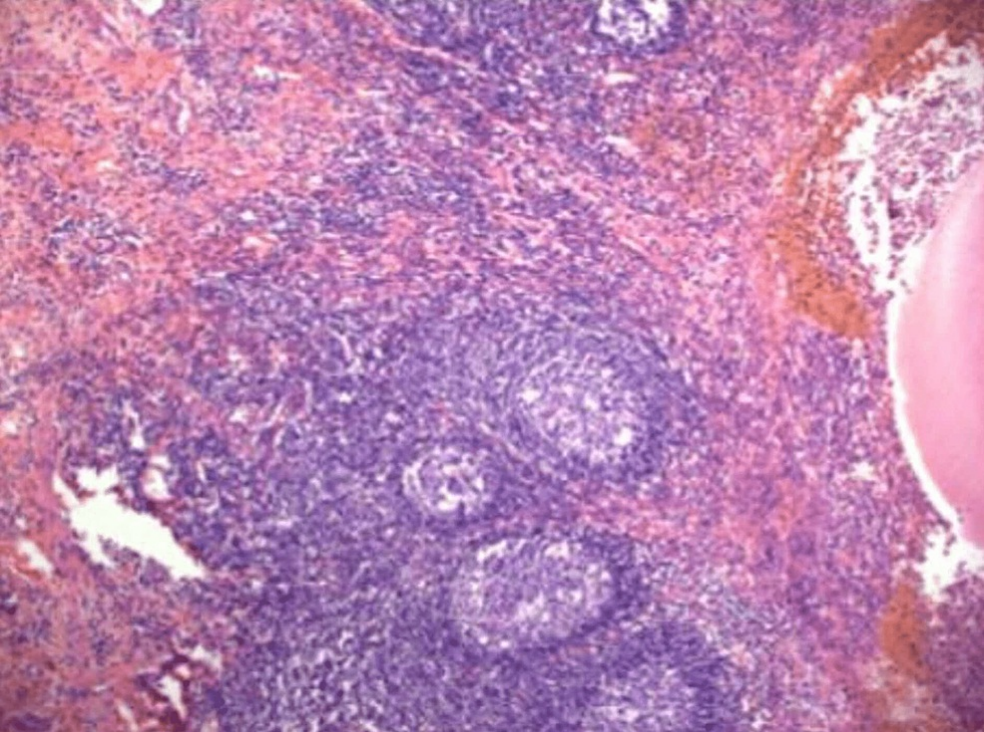
Appendicular wall with mucosal ulceration, lymphoid follicular hyperplasia and fibrinopurulent infiltrate.

A 53-year-old female patient presented to the emergency department with colicky abdominal pain of insidious onset for 24 hours in the right hemiabdomen, predominantly in the right iliac fossa associated with nausea and vomiting. She was admitted with a heart rate of 102 bpm, blood pressure of 77/47 mmHg, respiratory rate of 24 bpm, and temperature of 36.8 °C. She denies any history of importance. On physical examination, the findings were a soft depressible abdomen, painful to superficial and deep palpation in the right iliac fossa, with positive McBurney's point and Rovsing sign. As part of the approach, a plain tomography of the abdomen was requested, where perihepatic free fluid in the right paracolic gutter associated with free subdiaphragmatic air was observed in relation to pneumoperitoneum probably due to perforation of a hollow viscera (Figure 1); in the right iliac fossa, the cecal appendix was 0.37 cm (Figure 2). On arrival in the emergency department, her blood workup results were as follows: leukocytes 9.6 x10/uL, neutrophils 88%, lymphocytes 3%, and banded neutrophils 7%. With an Alvarado score of 6 points and the plain tomographic findings, it was decided to perform an exploratory laparotomy, thinking of acute appendicitis stage IV. Around 200 ml of free purulent fluid was found predominantly in the right paracolic gutter. Other findings include a pre-pyloric gastric perforation of 1 x 0. 5 cm (Figure 3) with an outflow of gastrobiliary fluid and a thickened and erythematous cecal appendix in the middle third, so gastric raffia, Graham patch placement, and appendectomy were performed. The surgical specimen was sent to pathology (Figure 4) where acute appendicitis with associated fibrinopurulent periapendicitis was reported. The specimen was 7.5 x 1 cm with intact walls, a gray-to-white surface with fibrinopurulent plaques, and a 0.4 cm wall with punctiform light and hemopurulent secretion. During the patient's hospital stay, she remained stable, with adequate symptomatology control. She was covered with antimicrobial treatment based on ertapenem and fluconazole and was released 96 hours after her admission, stable and making good progress.

## Discussion

VS is a rare entity; however, it should be kept in mind when assessing a patient with abdominal pain, since a misdiagnosis may not account for gastric or duodenal perforation and acute appendicitis, which can later cause complications if not properly treated, increasing morbidity and mortality. A peptic ulcer is a pathology caused by various medical conditions. The most common severe complication is perforation. Patients with this pathology come to the emergency room with abdominal pain and peritoneal irritation data; at this point, mortality can reach up to 20% [6]. The most common complication in patients with a perforated peptic ulcer is sepsis which is reported in up to 50% of patients [7]. The definitive diagnosis is made during the surgical exploration. Less than five of the reported cases were diagnosed before the surgical procedure and this was through computed tomography which is the test of choice for this entity [1]. Information that might be useful for the physician to suspect this pathology in the preoperative studies is the presence of free air in the retroperitoneum in case of a duodenal perforation, around the right kidney [2]. The right side's perirenal air causes the kidney to appear faded in imaging examinations, this is known as the "sign of the veiled right kidney" [1,4]. Management of this entity is surgical; a complete exploration of the abdominal cavity must be performed as in any laparoscopy or laparotomy. A vital clue observed during appendectomy could be a normal-appearing appendix with fluid in the abdomen [5] suggesting further exploration of the rest of the abdominal cavity. If a perforation site is located, it should be closed. Recently, a similar case of VS has been described, however, in this case, it was mimicking pancreatitis [3]. It is of utmost importance to remember these entities during the evaluation of patients with abdominal pain, as it will help to reduce morbidity and mortality should these cases occur.

## Conclusions

It is of utmost importance to recognize and remember the symptoms of Valentino syndrome since it can present in emergency rooms. If not managed appropriately, mortality due to peptic ulcer perforation can reach up to 20%. The management of this entity is surgical and locating the site of perforation should be correspondingly managed.

## References

[1] Noussios G, Galanis N, Konstantinidis S, Mirelis C, Chatzis I, Katsourakis A (2020). Valentino's syndrome (with retroperitoneal ulcer perforation): a rare clinico-anatomical entity. Am J Case Rep.

[2] Amann CJ, Austin AL, Rudinsky SL (2017). Valentino's syndrome: a life-threatening mimic of acute appendicitis. Clin Pract Cases Emerg Med.

[3] Arumugam B Jr, Giridharan B, R P, P N S (2022). Syndrome Valentino from a de novo aetiology - acute pancreatitis. Cureus.

[4] Mahajan PS, Abdalla MF, Purayil NK (2014). First report of preoperative imaging diagnosis of a surgically confirmed case of Valentino's syndrome. J Clin Imaging Sci.

[5] Jaboury IA (2020). Valentino syndrome: case report and review of literature. ANZ J Surg.

[6] Chung KT, Shelat VG (2017). Perforated peptic ulcer - an update. World J Gastrointest Surg.

[7] Amini A, Lopez RA (2019). Duodenal Perforation. https://www.ncbi.nlm.nih.gov/books/NBK553084/.

